# Development and Validation of Stability Indicating RP-HPLC Method for Voriconazole

**DOI:** 10.4103/0250-474X.58178

**Published:** 2009

**Authors:** A. B. Khetre, P. K. Sinha, Mrinalini C. Damle, R. Mehendre

**Affiliations:** Department of Pharmaceutical Chemistry, AISSMS College of Pharmacy, Pune-411 001, India; 1 Alkem Laboratories Ltd., Navi Mumbai-410 208, India

**Keywords:** RP-HPLC, stability indicating, validation, voriconazole

## Abstract

This study describes the development and validation of stability indicating HPLC method for voriconazole, an antifungal drug. Voriconazole was subjected to stress degradation under different conditions recommended by International Conference on Harmonization. The sample so generated was used to develop a stability-indicating high performance liquid chromatographic method for voriconazole. The peak for voriconazole was well resolved from peaks of degradation products, using a Hypersil C18 (250×4.6 mm) column and a mobile phase comprising of acetonitrile: water (40:60, v/v), at flow rate of 1 ml/min. Detection was carried out using photodiode array detector. A linear response (r > 0.99) was observed in the range of 5-25 μg/ml. The method showed good recoveries (average 100.06%) and relative standard deviation for intra and inter-day were ≤ 1.5 %. The method was validated for specificity and robustness also.

Voriconazole, chemically is (αR,βS)-α-(2,4-Difluorophenyl)-5-fluoro-β-methyl-α-(1H-1,2,4-triazol-1-yl-methyl)-4-pyrimideethanol[1]. It is used as an antifungal agent. Its primary mode of action is by inhibition of the fungal cytochrome P450-dependent 14α-sterol demethylase, an essential enzyme in ergosterol biosynthesis. Literature survey revealed many analytical methods for its estimation. Voriconazole has been quantitatively assayed in biological fluids by HPLC[2–8]. Determination of drug in pharmaceutical dosage form has been reported by methods that includes spectrophotometric and chromatographic techniques as well[9–10]. No useful method for the analysis of voriconazole in presence of its degradation products is yet reported.

The international Conferences on Harmonization (ICH) guideline[11] entitled stability testing of new drug substances and products requires that stress testing be carried out to elucidate the inherent stability characteristics of the active substance. The aim of this work was to develop stability indicating method for determination of voriconazole in presence of its degradation products. In the present study, stress degradation of voriconazole was effected by hydrolysis under acidic, basic, neutral conditions, oxidation with H_2_O_2_, dry heat degradation and photo degradation. The developed RP-HPLC method was validated following the ICH guidelines[12].

## MATERIALS AND METHODS

Voriconazole was kindly provided by Alkem Laboratories Ltd., Mumbai, India. Hydrochloric acid (AR), sodium hydroxide (AR) and hydrogen peroxide (AR) were purchased from Loba Chemie Pvt. Ltd., Mumbai, India. Acetonitrile and water were purchased from Merck, Mumbai, India. Voriconazole tablets were procured from a local pharmacy. Jasco HPLC system (2000 series) comprising of Jasco PU-2080 plus intelligent pump, Jasco MD- 2010 plus multiwavelength detector and Rheodyne 7725i injector fitted with 20 μl capacity loop was used in the study. Separations and quantitation were done using Hypersil C18 (250×4.6 mm) column.

### Chromatographic conditions:

The mobile phase was prepared by mixing acetonitrile and water in a ratio of 40:60 v/v. The mobile phase was filtered using 0.45 μm filter and degassed by ultrasonic vibrations prior to use. The flow rate was 1 ml/min. All determinations were performed at ambient temperature. An accurately weighed sample (10 mg) of voriconazole was transferred to a 10 ml volumetric flask and dissolved in acetonitrile to obtain a solution of strength 1000 μg/ml. One millilitre of this solution was then transferred in 10 ml volumetric flask and made up the volume was made up to the mark with mobile phase. This gave the standard stock solution of 100 μg/ml.

**Fig. 1:**
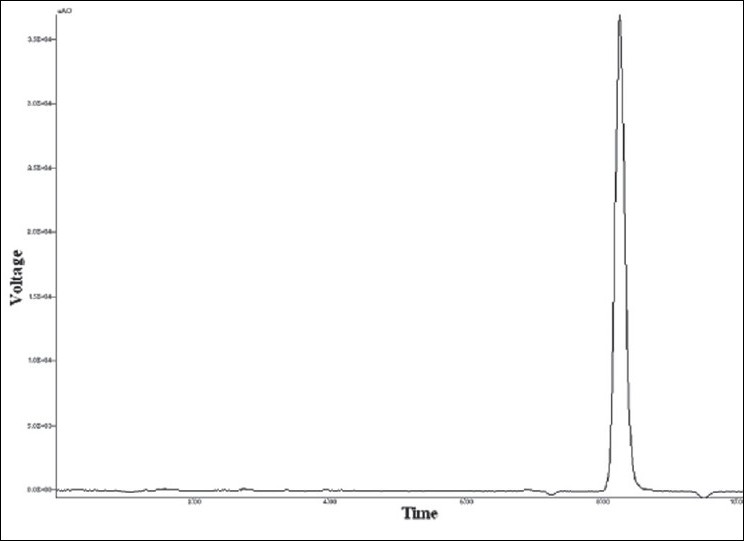
Standard chromatogram of voriconazole (15 μg/ml)

From the standard solution of voriconazole (100 μg/ml), appropriate dilutions were prepared in mobile phase to get final concentrations in the range of 5-25 μg/ml. These standard solutions were analyzed in five replicates. The peak areas were plotted against concentration and the data was subjected to linear regression. The standard chromatogram of voriconazole is shown in fig. 1

### RP-HPLC Assay procedure:

Twenty Tablets, each containing 200 mg voriconazole were weighed and finely powdered. A quantity of powder equivalent to 10 mg was weighed and transferred to 10 ml volumetric flask containing about 5 ml acetonitrile, ultrasonicated for 10 min and then the volume was made up to 10 ml with acetonitrile. The solution was filtered using Whatmann filter paper No. 41. From the filtrate appropriate dilutions were made in mobile phase to obtain concentration in the range of 5 to 25 μg/ml. The tablet sample solution was injected and chromatogram was obtained. The peak area of the voriconazole was calculated. Using the regression equations and peak areas of the sample, the amount of voriconazole in the sample was calculated. The amount of voriconazole per tablet was thus found.

### Method Validation:

Linearity of the method was studied by injecting five concentrations of the drug prepared in the mobile phase in the range of 5-25 μg/ml into the HPLC system and noting the peak areas. Precision of the method was demonstrated by interday and intraday variation studies. In the intraday studies analyses of three different concentrations of the drug was repeated thrice in a day. In the inter day variation studies analyses of three different concentrations of the drug was repeated on three consecutive days. Accuracy of the method was determined by recovery experiments. The recovery studies were carried out at three levels of 80, 100 and 120% and percent recovery was calculated.

The detection limit of an individual analytical procedure is the lowest amount of analyte in a sample, which can be detected but not necessarily quantitated as an exact value. The quantitation limit of an individual analytical procedure is the lowest amount of analyte in a sample, which can be quantitatively determined with suitable precision and accuracy. These were calculated using the formula involving standard deviation of response and slope of calibration curve.

Robustness of the method was determined by carrying out the analysis under conditions during which mobile phase composition (concentration of acetonitrile was varied by ±1%), and flow rate (varied by ±0.05 ml/min) were altered and the effect on the area of peak of interest and retention times was noted. Commonly used tablet excipients were subjected to chromatographic analysis and were observed for any of the interfering peaks at the retention time of voriconazole. Specificity was also confirmed by peak purity values given by PDA detector. Value more than 900 indicates non-interference by matrix components.

### Stress degradation by hydrolysis under acidic conditions:

To 7.5 ml of stock solution of voriconazole 2.5 ml of 3 N HCl was added in 25 ml of volumetric flask and made up the volume to the mark with mobile phase. Volumetric flask was kept at normal condition for 150 min. After each 30 min time interval 5 ml solution was pipette out from this flask, neutralized and diluted with mobile phase in order to make the volume up to 10 ml. This solution was injected in stabilized chromatographic condition. For the blank, 0.5 ml solution of 3 N HCl and 0.5 ml solution of 3 N NaOH diluted with mobile phase in 10 ml of volumetric flask.

### Stress degradation by hydrolysis under alkaline conditions:

To 7.5 ml of stock solution of voriconazole 2.5 ml of 0.1 N NaOH was added in 25 ml of volumetric flask and made up the volume to the mark with mobile phase. Volumetric flask was kept at normal condition for 150 min. After each 30 min time interval pipetted out 5 ml solution from this flask, neutralized and diluted with mobile phase in order to make the volume up to 10 ml. This solution injected in stabilized chromatographic condition. For the blank, 0.5 ml solution of 0.1 N HCl and 0.5 ml solution of 0.1 N NaOH diluted with mobile phase in 10 ml of volumetric flask.

### Stress degradation by hydrolysis under neutral conditions:

To 5 ml of stock solution of voriconazole, 45 ml of water was transferred in to 100 ml of round bottom flask and was refluxed for 1 h on boiling water bath. After refluxing, cooled solution to room temperature and again made up the volume up to the 50 ml and then injected in stabilized chromatographic condition.

### Dry heat-induced degradation:

Voriconazole sample was taken in petriplate and kept in an oven maintained at 70° temperature for 48 h. Ten milligrams of the above sample was dissolved in and diluted with acetonitrile in order to make the volume up to 10 ml. From this solution appropriate dilution was made using mobile phase and injected in stabilized chromatographic condition.

### Oxidative degradation:

To 1.5 ml of stock solution of voriconazole, 1 ml of 30% w/v of H_2_O_2_ was added in 10 ml of volumetric flask and made up the volume up to the mark with mobile phase. Volumetric flask kept at room temperature for 15 min. For the blank, 1 ml of 30% w/v of H_2_O_2_ was kept at normal conditions for overnight in 10 ml of volumetric flask. Both solutions were heated on boiling water bath to remove the excess of hydrogen peroxide. Finally made up the volume to 10 ml by mobile phase, and then injected in stabilized chromatographic condition.

### Photolytic degradation:

Sample of voriconazole was exposed to both cool white fluorescent and near ultraviolet lamp in photostability chamber providing illumination of not less than 1.2 million lux hours. Ten milligrams sample was dissolved in acetonitrile and volume made up to 10 ml. From this solution appropriate dilution was made using mobile phase and injected in stabilized chromatographic condition.

### Accelerated stability study of tablets:

According to ICH guidelines, an accelerated stability study has to be carried out on the pharmaceutical dosage form at 40±2°/75±5% RH. During the present study, sample of tablets containing voriconazole was subjected to accelerated stability study. The tablets were placed in stability chamber at 40°/75% RH for one month. Ten tablets were withdrawn at end of first, second, third and fourth weeks. After withdrawal, the tablets were crushed and a quantity of powder equivalent to 10 mg of voriconazole was weighed and transferred to 10 ml volumetric flask containing about 6 ml acetonitrile, ultrasonicated for 10 min and then the volume was made up to 10 ml with acetonitrile. The solution was filtered using Whatmann filter paper No. 41. From the filtrate appropriate dilutions were made in mobile phase to obtain concentration of 15 μg/ml. The tablet sample solutions were injected and chromatogram was obtained. The peak areas of the drugs were obtained.

## RESULTS AND DISCUSSION

The assay was calculated from the equation of regression line. The percentage of drug found in formulation was 99.81±0.21%. The results of analysis show that the amount of drug was in good agreement with the label claim of the formulation. The data obtained in the calibration experiments when subjected to linear-regression analysis showed a linear relationship between peak areas and concentrations in the range of 5-20 μg/ml for voriconazole. The equation of the regression line is y = 24537×+27363 (r^2^ = 0.9923).

The developed method was found to be precise as the % RSD values for intra-day and inter-day precision studies were found to be less than 2%. Good recoveries (99.74-100.39%) of the drug were obtained at each added concentration, indicating that the method was accurate. Commonly used tablet excipients were subjected to chromatographic analysis and it was observed that there was no interfering peak at the retention time of voriconazole. Specificity was also indicated by the resolution of voriconazole peak from the peaks for degradation product. The peak purity profile by PDA detector confirmed the specificity. The LOD and LOQ were found to be in the submicrogram level for voriconazole, thus indicating sensitivity of the method. During robustness check, the peak areas of drug were found to vary in the range ±0.0093% whereas the retention time was found to vary in the range 8.231 to 8.268 min. The method was thus found to be robust since the monitored parameters i.e. the areas of peaks of interest and retention time were not significantly affected when checked by varying the parameters like mobile phase composition and flow rate. Summary of validation parameters of proposed HPLC method is shown in Table 1.

**TABLE 1:** SUMMARY OF VALIDATION

Parameter	Result
Linearity indicated by coefficient of correlation (r^2^)	0.9923
Precision indicated by % RSD	< 2%
Accuracy indicated by % Recovery	99.74-100.39
Limit of Detection	0.1841 μg/ml
Limit of Quantification	0.5581 μg/ml
Range	5-25 μg/ml
Linear regression equation	y = 24537× + 27363

**Fig. 2:**
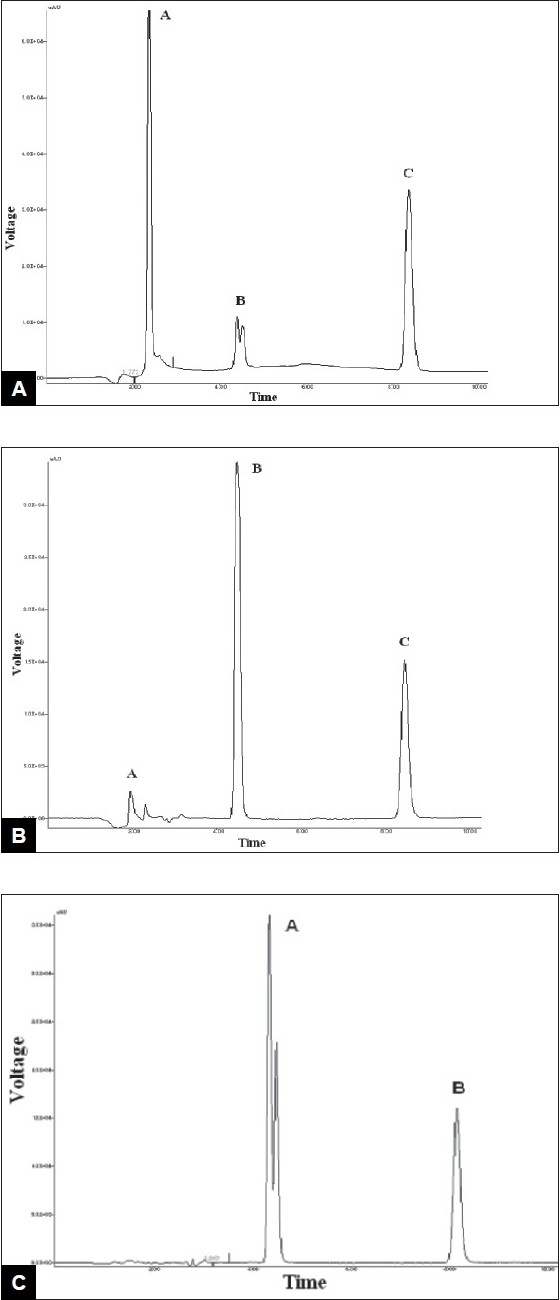
Chromatogram of voriconazole subjected to hydrolytic conditions 2A: acid hydrolysis under acidic condition, 2B: hydrolysis under basic condition, 2C: hydrolysis under neutral condition.

**Fig. 3:**
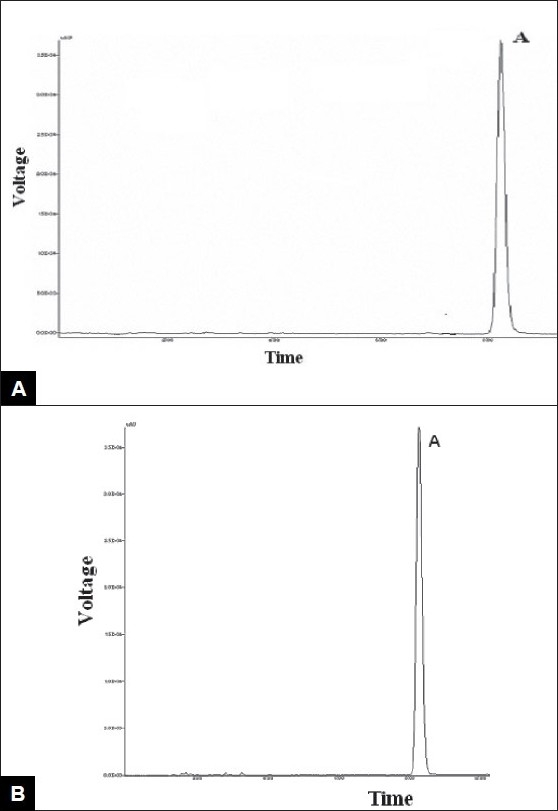
Chromatogram of voriconazole subjected to heat and light 3A: upon exposure to heat and 3B: upon exposure to light.

**Fig. 4:**
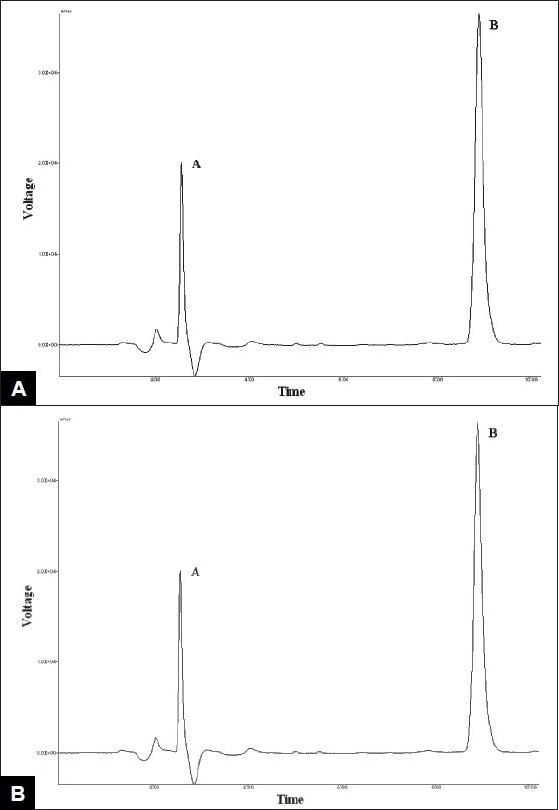
Chromatogram of voriconazole subjected to oxidative conditions 4A: 30% H2O2 (15 min) degradation, 4B: 30% H2O2 (20h) degradation.

Voriconazole drug was found to degrade under acidic condition. When voriconazole was treated with 3 N HCl and sample was withdrawn at an interval of 30, 60, 90, 120 and 150 min the result presented in Table 2 was obtained and the typical chromatogram obtained after 60 min is shown in fig. 2a. The degradation reaction was more intense and quicker in alkaline condition. When voriconazole was treated with 0.1 N NaOH and sample was withdrawn at an interval of 30, 60 and 90 minutes the result presented in Table 2 was obtained and the typical chromatogram obtained after 30 min is shown in fig. 2b. Voriconazole was found to get hydrolyzed, upon refluxing with water on boiling water bath, which was evident as the peak area for voriconazole in the degradation sample was found to be less by 56.92% compared to the corresponding peak area for zero time samples. Chromatogram of voriconazole sample degraded under neutral hydrolytic condition is shown in fig. 2c. When voriconazole was exposed to heat, peak area for voriconazole in the degradation sample was found to be less by 0.24% compared to the corresponding peak area for zero time samples. No additional degradation peaks were detected. Fig. 3a shows chromatogram of voriconazole sample exposed to dry heat. When voriconazole was exposed to light source as per ICH guidelines, the voriconazole content exhibited slight decrease, but no additional peaks were detected. Fig. 3b shows chromatograms of voriconazole samples degraded under Photolytic condition. Upon treatment of voriconazole with 1 ml of 30% w/v H_2_O_2_ at normal condition for 15 min and 5 ml of 30% w/v H_2_O_2_ at normal condition for 24 h, no additional peaks were detected but peak area for voriconazole was found to be less by 1.17% and 5.40% compared to the corresponding peak area for zero time samples. Figs. 4a and 4b show chromatograms of voriconazole samples degraded under oxidative condition.

**TABLE 2:** RESULT OF STRESS DEGRADATION STUDIES OF VORICONAZOLE

Condition	Time (Min.)	% Degradation	Retention time of degradation Products (min.)
0.1 N NaOH	30	55.58	4.45
	60	78.75	4.44
	90	100	4.44
3 N HCl	30	11.60	4.41
	60	16.92	4.41
	90	25.11	4.42
	120	29.99	4.45
	150	35.06	4.44
30% H_2_O_2_(1 ml)	15	1.17	None Detected
30% H_2_O_2_(5 ml)	20 h	5.40	None Detected
Dry Heat 70°	48 h	0.24	None Detected
Photolytic	-	4.68	None Detected
Neutral (Reflux)	1 h	56.92	4.3

**Fig. 5:**
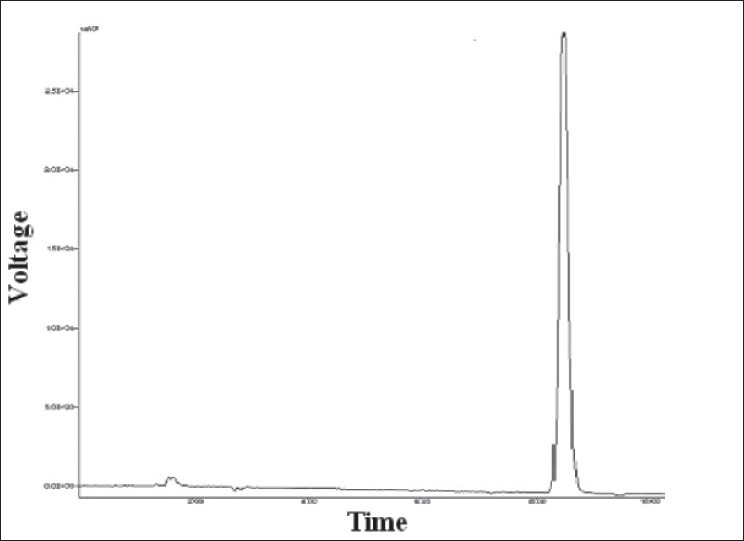
Chromatogram of voriconazole tablets (15 μg/ml).

Fig. 5 shows the initial chromatogram of voriconazole tablets. When the marketed tablets without primary packaging were subjected to accelerated stability study at 40°/75% RH and withdrawn at an interval of first, second, third and fourth week, the % degradation found was 18.21, 26.03, 36.46 and 36.92%, respectively. There was no peak for product of degradation. This indicates importance of careful selection of primary packaging material so as to protect it from moisture.

Thus the study shows that voriconazole undergoes degradation in acidic, alkaline and neutral hydrolytic conditions whereas it is relatively stable when exposed to dry heat, oxidation and photolytic conditions. A stability-indicating method was developed, which resolved all the degradation products formed under variety of conditions. The degradation products formed under acid, alkali and neutral hydrolysis condition are same which is confirmed from UV spectrum of the degraded product. The method proved to be simple, accurate, precise, specific and selective. Hence it may be used to assay of the product during stability studies.
